# Trans-umbilical Single-Site Plus One Robotic Assisted Surgery for Choledochal Cyst in Children, a Comparing to Laparoscope-Assisted Procedure

**DOI:** 10.3389/fped.2022.806919

**Published:** 2022-02-25

**Authors:** Shan Lin, Jianglong Chen, Kunbin Tang, Yufeng He, Xinru Xu, Di Xu

**Affiliations:** ^1^Department of Pediatric Surgery, Fujian Provincial Hospital, Fuzhou, China; ^2^Shengli Clinical Medical College of Fujian Medical University, Fuzhou, China

**Keywords:** single-site plus one, robot, laparoscopy, choledochal cyst, pediatric

## Abstract

**Objective:**

We introduce the trans-umbilical single-site plus one robotic-assisted surgery for the treatment of pediatric choledochal cyst. Compare the intraoperative and postoperative outcomes between the new method and traditional laparoscopy-assisted procedure.

**Method:**

We retrospectively analyzed the clinical data of 51 children diagnosed with choledochal cysts and received surgery from June 2019 to December 2020 at our department. About 24 patients who underwent the robot-assisted procedure were selected as the R group, and 27 patients who underwent the laparoscope-assisted procedure were selected as the L group. We compare the intraoperative and postoperative outcomes between the two groups.

**Result:**

No significant differences were found in demographic information between the two groups (*P* > 0.05). The median total operative time, median port/trocar installation time, and median wound suture time of the R group were a little longer than the L group (217.63 ± 5.90 vs. 199.37 ± 5.13 min; 30.71 ± 3.18 vs. 6.11 ± 1.15 min; 30.79 ± 1.82 vs. 20.40 ± 3.12 min, respectively; *P* < 0.001). However, the R group had shorter choledochal cyst excision time and mean hepaticojejunostomy anastomosis time than the L group (52.04 ± 2.74 vs. 59.26 ± 3.23 min; 52.42 ± 2.72 vs. 60.63 ± 3.30 min, respectively, *P* < 0.001). The mean extracorporeal Roux-y jejunojejunostomy time of two groups has no remarkable difference (*P* > 0.05). The R group also had less mean volume of blood loss (7.04 ± 1.16 vs. 29.04 ± 18.21 mL; *P* < 0.001). The R group had a shorter indwelling time of gastric tube, anal exhaust time, water feeding time, solids feeding time, and hospital stay time than the L group (*P* < 0.05). The R group had a lower early complication rate than the L group (4.2 vs. 29.63%; *P* = 0.026). No statistical differences were identified between the two groups in late or any single complication (0.00 vs. 11.11%; *P* > 0.05).

**Conclusions:**

A resection of the choledochal cyst and a Roux-en-Y hepaticojejunostomy can be performed much more precisely by single-site plus one robotic-assisted surgery. Patients can achieve rapid recovery, and the umbilical incision is more concealed and beautiful. Combing the experience of single-site surgery with robot-assisted surgery, the operators can implement the technique in children safely and feasibly.

## Introduction

A choledochal cyst is a congenital anomaly of the biliary system, characterized by the dilatation of the intrahepatic or extrahepatic bile ducts and often associated with pancreaticobiliary maljunction. It is much more common in Asian countries than in western countries, with an incidence of 1,000 cases per million people (1). The incidence in females is four times higher than that in males (2). A choledochal cyst can be detected in the antenatal period using prenatal sonography and may present a variety of symptoms: abdominal pain, jaundice, or abdominal mass after birth (3). Minimally invasive surgery has become the mainstream with the advantages of cosmetic appearance and rapid postoperative recovery. Currently, minimally invasive surgery for choledochal cyst includes laparoscopy-assisted and robot-assisted procedures. Since 2015, our department has been working to promote a minimally invasive surgical technique for choledochal cysts. In 2015, we carried out a modified trans-umbilical single-incision laparoscopic technique and achieved satisfactory postoperative outcomes (4). In 2018, we implemented a quadruple-channel puncture device instead of a traditional laparoscopic trocar to complete single-site surgery, which was more convenient for an operator while still maintaining the excellent outcome. In 2020, after importing the da Vinci Robotic Surgical System (Intuitive Surgical, Sunnyvale, CA, USA), we combined the robot system with a quadruple-channel puncture device to invent trans-umbilical single-site plus one robotic-assisted choledochal cyst excision and hepaticojejunostomy. The new modified technique not only makes the procedure more sophisticated through a robot system, but it also retains the minimally invasive appearance of trans-umbilical single-site surgery. Therefore, we conducted a retrospective study to investigate the safety and effectiveness of laparoscopic procedures and trans-umbilical single-site plus 1 robotic- assisted procedure in pediatric choledochal cyst excisions.

## Materials and Methods

### Patients

From June 2019 to December 2020, we attempted a resection of the cyst and a Roux-en-Y hepaticojejunostomy anastomosis in 51 children at our department. We retrospectively analyzed the clinical data of these patients. About 24 patients who underwent the robot-assisted procedure were selected as the R group, and 27 patients who underwent the laparoscopy-assisted procedure were selected as the L group. Among the children, there were 10 patients who were detected in the antenatal period; 3 of them present with jaundice after birth and underwent surgery early in the month. The minimum operation age was 3 months old.

During the study period, all patients were diagnosed by magnetic resonance cholangiography. The age, weight, sex, symptom, follow-up time, cyst diameter, and Todani modification of the Alonso–Lej classifications between the two groups were similar (Table 1).

**Table 1:** Demographic information.

	**R Group**	**L Group**	***t/X^2^*/F**	***P***
N	24	27		
Age (month)	30.13 ± 13.88 (3~54)	33.56 ± 11.56 (8~60)	−1.146	0.255
Sex			*X2*	
Boy	9	11		
Girl	15	16	0.056	0.813
Weight (kg)	12.99 ± 3.42 (5–16.3)	13.72 ± 2.63 (7–17.4)	−0.855	0.397
Cyst diameter (mm)	37.54 ± 7.42 (15–50)	36.89 ± 5.82 (18–48)	0.351	0.727
Following time (month)	13.38 ± 2.02 (11–18)	14.04 ± 3.18 (9–18)	−0.875	0.386
Symptoms			*X2*	
Jaundice	4	2		
Abdominal pain	10	8		
Abdominal mass	10	17	2.54	0.281
Todani modification of the Alonso–Lej classifications			F	
Ia	14	15		
Ib	2	1		
Ic	5	6		
IVa	3	4		
IVb	0	1	1.552	1.00

The inclusion criteria: With clinical symptoms or abdominal ultrasonography showing suspicion of biliary dilatation diagnosed by magnetic resonance cholangiography as choledochal cysts. Acute cholangitis was conservative treatment until abnormal hepatic function and coagulation disorders were corrected.

The exclusion criteria: A history of abdominal surgery due to Choledochal cyst (CC) rupture or punching. Comorbidities such as other gastrointestinal anomalies.

Informed of both the advantages and disadvantages of the two operative methods, the patient's parents selected the approach according to their preferences and economic capabilities. All operations were done by the same term of experienced surgeons.

### Surgical Techniques

Intestinal preparation commonly includes two parts: preoperative intestinal lavage and antibiotic prophylaxis. After the application of general anesthesia and endotracheal intubation, all patients were placed with a catheter and gastric tube and put in the supine position with their head elevated 30° and at an incline of 30°-45° to the left side. We created carbon dioxide pneumoperitoneum with a pressure of 10–12 mmHg and a flow of 4 mL/min by using a Veress needle.

### Trans-umbilical Single-Site Plus 1 Robotic-Assisted Approach

#### The Location of Ports

**Figure 1:**
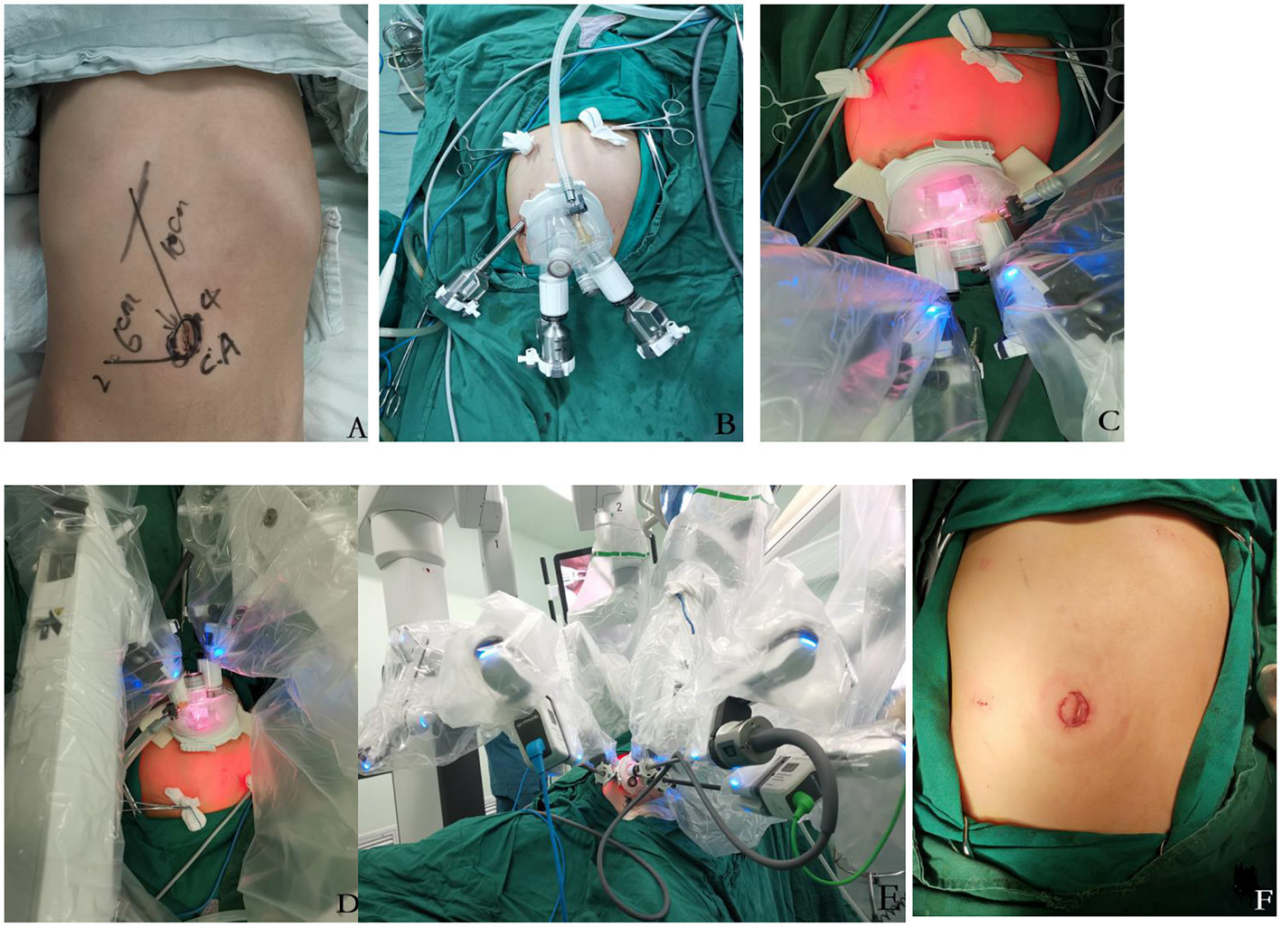
**(A–F) (A,B)** the design location of quadruple-channel puncture device and port II. **(C–E)** after the docking of a robotic system, the distance between each robotic port was about 4 cm, which was effectively avoiding instrument collision. **(F)** the cosmetic appearance of the wound of trans-umbilical single-site plus 1 robotic-assisted approach.

Although the da Vinci system we used had four robotic arms, we only used three [three-dimensional (3D) camera port III, operating port II, operating port IV] and introduced a quadruple-channel puncture device to improve cosmetic appearance. Making a 2.5–3 cm curved incision along the edge of the umbilical to place a quadruple-channel puncture device, whose channels would be used to put an 8 mm 3D camera port III, an 8 mm operating port IV and be used as an assistant port in a subsequent operation. The other 8 mm incision was made 6 cm on the right-side robotic operating port II. The location of single port, the scenes of robotic after docking and the cosmetic appearance have been showed in Figure 1.

#### Extracorporeal Roux-en-Y Jejunojejunostomy

The surgeon used laparoscopic forceps and a robotic 3D camera to explore the abdominal cavity and identified the Treitz ligament, 25 cm from which to be the planned incision site. Then, he removed the quadruple-channel puncture device but kept the baseplate, grasped the jejunum, and exteriorized it through the baseplate for extracorporeal Roux-en-y jejunojejunostomy. Transecting it at the planned site with a linear stapler, folding the proximal jejunum, side to side with the distal jejunum 20 cm from the transection point with an overlap of about 4 cm, he fied the two ends each with a stitch; on the overlapped sides, he made a 1 cm opening each along the lateral wall at the anti-mesenteric border, placed the cartridge seat and anvil, one in each opening, and completed the anastomosis of the overlapping jejunum; then, he sutured the opening site of the anti-mesenteric border with 5–0 Vicryl sutures to complete the side-to-side hepaticojejunostomy anastomosis. He then placed the jejunum back in order in the peritoneal cavity.

#### Sequential Traction Suture to Complete the Surgery

The quadruple-channel puncture device was reset, the pneumoperitoneum was re-established, and the da Vinci system was docked. Traction sutures were inserted through the abdominal wall below the xiphoid and the right hypochondrium; suture the round ligament and the tissue of the gallbladder fundus, and then be pulled out from the inserted position. In this way, the liver was pulled to the abdominal wall and stabilized at two ends, which enlarges the operative field and facilitates optimal visualization of the porta hepatis.

The cyst was loosened from the gallbladder and from the duodenum with an ultrasonic scalpel and detached; the cystic arteries were ligated with clamps, then the gallbladder was disassociated to the junction of the cystic duct and the common bile duct, the peritoneum was detached from the anterior CC wall to expose the cyst, and the cyst was then punctured and drained of bile. In a similar manner, the posterior cyst wall was separated with the ultrasonic scalpel tightly kept to the cyst wall (being careful not to damage the portal vein) and the cyst transected. The duodenum was pulled with forceps, the ultrasonic scalpel kept to the cyst wall, and the CC wall was separated toward the thinning distal end of the cyst converging with the pancreatic duct; the distal end of the common bile duct was clipped with a hemo-lock, and then the distal cyst wall was resected. In a similar fashion, the proximal part of the cyst wall was separated with an ultrasonic scalpel from its normal junction with the hepatic duct; the opening of the left and right hepatic duct was identified, and then the proximal cyst was transversely dissected and resected, leaving 10 mm of common hepatic bile duct for the hepaticojejunostomy anastomosis.

**Figure 2:**
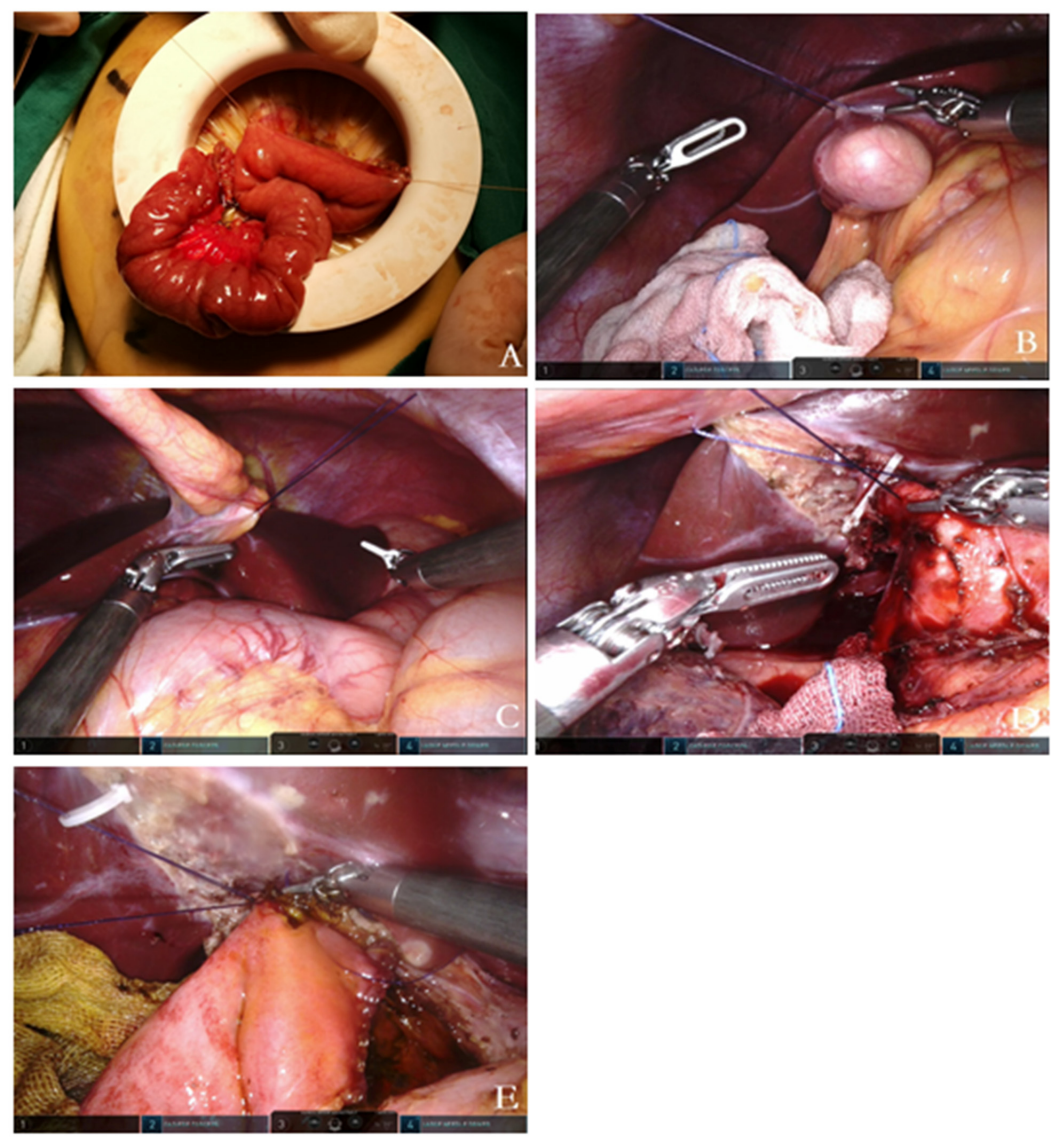
**(A)** Extracorporeal Roux-en-y jejunojejunostomy; the baseplate of the device can serve as a container. **(B–E)** Sequential traction suture; **(B)** stitch and lift the gallbladder fundus; **(C)** stitch and lift the round ligament; **(D)** stitch and lift the common bile duct before dissecting the choledochal cyst; **(E)** stitch and lift the common hepatic before the hepaticojejunostomy.

The vessel-free area to the right of the colon artery was opened up; a suitable length of the Roux limb was placed *via* the tunnel behind the colon and then to the hilum, securing its position. The jejunal wall was cut open at the anti-mesenteric edge, 5–0 Vicryl sutures were inserted through the right hypochondrium and tape the jejunum to the right side of the common hepatic duct, and then the sutures were retracted through the abdominal wall to pull up the common hepatic duct. The lifting and traction were adjusted to facilitate anastomosis. Then, continuous stitches were used to tape the posterior wall of the common hepatic duct to the back wall of the jejunum with 4–0 absorbable barbed sutures. In the same manner, an anastomosis was performed of the anterior wall of the common hepatic duct and the front wall of the jejunum. The peritoneal cavity was rinsed, and then the drainage tube was guided through the operating trocar hole on the right of the umbilicus to the appropriate place. We had to confirm that there was no hemorrhage, the pressure was released, the trocar was removed, and the incision was sutured closed. The surgical steps of extra-abdominal jejunojejunostomyLand laparoscopic cholangioenterostomy by robot were showed in Figure 2.

### Laparoscope-Assisted Approach

#### The Location of Trocar

A 1.2 cm incision was made to insert a 12 mm camera trocar below the umbilical, and this was used to introduce a 30°5 mm laparoscope into the abdomen. Two 5 mm operating trocars are inserted in the right upper quadrant and left upper quadrant, which were about 6 cm away from the umbilical. A 5 mm assistant trocar was placed between the camera trocar and the left operating trocar.

#### Enlarge Umbilical Incision to Achieve Extracorporeal Roux-y Jejunojejunostomy

Flip the transverse colon up, and identify the Treitz ligament, 25 cm from which to be the planned incision site. Then, enlarge the umbilical incision to 3 cm and take out the jejunum to complete the side-to-side hepaticojejunostomy anastomosis as mentioned above.

#### Sutured Umbilical Wound and Re-established Pneumoperitoneum to Complete the Surgery

The jejunum was placed back in order in the abdominal cavity. The umbilical wound was sutured to fit a 12 mm port. We re-established pneumoperitoneum and accomplished the surgery.

## Postoperative Progress

We removed the gastric tube postoperative when the volume of drainage fluid was lower than 10 mL. Then, water was given first, followed by a liquid diet and then a soft diet. After all, the diets are able to be consumed by the patient with normal urination and defecation. If there is no fever of undetermined origin, abdominal pain, or other complications and preoperative ultrasonography is normal, only then is discharge considered.

## Follow-Up And Data Collection

The data of all patients were collected.

Demographic information included the following: age, weight, sex, symptom, follow-up time, cyst diameter, Todani modification of the Alonso–Lej classifications. Operative details included the following: total operation time, port/trocar installation time, extracorporeal Roux-y jejunojejunostomy time, choledochal cyst excision time, hepaticojejunostomy anastomosis time, wound suture time, volume of blood loss, and intra-operative blood transfusion. Postoperative outcomes included the following: anal exhaust time, water and solids feeding time, hospital stay time, indwelling time of gastric tube, ultrasound-guided puncture drainage rate, and complications. Early complications were defined as complications that occurred within 30 days postoperatively, such as upper respiratory infection, anastomotic bleeding, wound infection, and bile leakage. Late complications referred to cholangitis, residual cyst, and distal lithiasis.

## Statistical Analyses

Continuous variables in this study were presented as median (interquartile range) or mean ± standard deviation (SD) according to their distribution. Significant differences between the two groups were tested by the *Fisher* exact test for categorical variables and the independent-samples test for continuous variables. All the data and analyses were performed by Statistical Product and Service Solutions (SPSS) 25.0, and statistical significance was defined as *P* < 0.05.

## Result

This study contains 51 patients, including 24 patients who underwent robot-assisted procedures (the R group) and 27 patients who underwent laparoscopy-assisted procedures (the L group). In the R group, the average following time was 13.38 ± 2.02 months. The male-to-female ratio was 3:5, and the median age of the patient was 30.13 ± 13.88 months with a mean weight of 12.99 ± 3.42 kg. The most common symptoms were jaundice, abdominal pain, abdominal mass (16.67, 41.67, and 41.67%, respectively). The cyst size in the series was 37.54 ± 7.42 cm. The Todani modification of the Alonso–Lej classifications were Ia, Ib, Ic, IVa, and IVb (58.3, 8.3, 20.83, 12.5, and 0%). In the L group, the average following time was 14.04 ± 3.18 months. The ratio of male to female was 11:16, and the median age of the patient was 33.56 ± 11.56 months with a mean weight of 13.72 ± 2.63 kg. The most common symptoms were jaundice, abdominal pain, abdominal mass (7.4, 29.63, and 62.96, respectively). The cyst size in the series was 36.89 ± 5.82 cm. The Todani modification of the Alonso–Lej classifications were Ia, Ib, Ic, IVa, and IVb (55.56, 3.7, 22.22, 14.81, and 3.7%). No significant differences were found in the demographic information between the two groups (*P* > 0.05) (Table 1).

The median total operative time, median port/trocar installation time, and median wound suture time of the R group were a little longer than in the L group (217.63 ± 5.90 vs. 199.37 ± 5.13 min; 30.71 ± 3.18 vs. 6.11 ± 1.15 min; 30.79 ± 1.82 vs. 20.40 ± 3.12 min, respectively; *P* < 0.001). However, the R group had a shorter choledochal cyst excision time and mean hepaticojejunostomy anastomosis time than the L group (52.04 ± 2.74 vs. 59.26 ± 3.23 min; 52.42 ± 2.72 vs. 60.63 ± 3.30 min, respectively; *P* < 0.001). The mean extracorporeal Roux-y jejunojejunostomy time of the two groups has no remarkable difference (*P* > 0.05). The R group also had less mean volume of blood loss (7.04 ± 1.16 vs. 29.04 ± 18.21 mL; *P* < 0.001). There were two patients who had an intra-operative blood transfusion in the L group but showed no significant difference (*P* > 0.05) (Table 2).

**Table 2:** Operative details.

	**R Group**	**L Group**	***t***	***P***
N	24	27		
Total operation time (min)^*^	217.63 ± 5.90 (207~233)	199.37 ± 5.13 (189~210)	11.824	<0.001
Port/Trocar installation time (min)^*^	30.71 ± 3.18 (25~37)	6.11 ± 1.15 (5~9)	37.517	<0.001
Extracorporeal Roux-en-y jejunojejunostomy time(min)^*^	52.67 ± 3.53 (48~60)	52.96 ± 3.88 (48~61)	−0.284	0.778
Choledochal cyst excision time (min)^*^	52.04 ± 2.74 (45~56)	59.26 ± 3.23 (52~64)	−8.55	<0.001
Hepaticojejunostomy anastomosis time^*^ (min)	51.41 ± 2.72 (47~56)	60.63 ± 3.30 (55~67)	−10.787	<0.001
Wound suture time (min)^*^	30.79 ± 1.82 (28~35)	20.41 ± 3.12(17~32)	14.29	<0.001
Volume of blood loss (min)	7.04 ± 1.16 (6~10)	29.04 ± 18.21 (15~100)	−5.900	<0.001
Intra-operative blood transfusion	0	2	F	
	24	25		0.492

^*^Total operation time (min) was the period from incising the skin at the beginning to finishing the wound closure at the end of the operation.

^*^Port/Trocar installation time (min): The R group's port installation time was defined as the time of making the curved incision along the umbilical to introduce a quadruple-channel puncture device to place the robotic ports IV and III and making another 8 mm incision to place the robotic port II, and finally docking the robotic system. The L group's trocar installation time was referred to as the time to make incisions to put one 12 mm trocar and three 5 mm trocars and connect the laparoscopic system.

^*^Extracorporeal Roux-en-y jejunojejunostomy time (min) was calculated from identifying and grasping the jejunum and exteriorizing it through the baseplate of the quadruple-channel puncture device or enlarged umbilical wound to finishing extracorporeal Roux-en-y jejunojejunostomy and then returning the jejunum into the abdominal cavity.

^*^Choledochal cyst excision time (min) was the time of fully disassociating and excising the gallbladder and choledochal cyst.

^*^The hepaticojejunostomy anastomosis time was counted from producing a tunnel at the vessel-free area of the right colon mesentery, traversing the Roux limb to the hilum, to finish the hepaticojejunostomy anastomosis.

^*^Wound suture time (min) : The R group's wound suture time was the time of suturing the curved incision along the umbilical and another 8 mm incision on the right side of the umbilical.

*The L group's wound suture time included enlarging and suturing umbilical incision at the extracorporeal Roux-en-y jejunojejunostomy stage and finally closing the wound at the end of the operation*.

The R group had a shorter indwelling time of the gastric tube, anal exhaust time, water feeding time, solids feeding time, and hospital stay time than the L group (*P* < 0.05). The R group had a lower early complication rate than the L group (4.2 vs. 29.63%; *P* = 0.026). In the L group, one child had anastomotic bleeding accompanied by bile leakage and one child had bile leakage; both of them underwent ultrasound-guided catheter drainage and received conservative treatments. Three children had upper respiratory infections. Three children had wound infections. Only one child in the R group had an upper respiratory infection. No statistical differences were identified between the two groups in late or any single complication (0.00 vs. 11.11%; *P* > 0.05). Two children who developed cholangitis received antibiotic therapy; one child developed stenosis of hepatoenteric anastomosis and received hepaticojejunostomy anastomosis again (Table 3).

**Table 3:** Postoperative outcomes.

	**R Group**	**L Group**	***t***	***P***
N	24	27		
Indwelling time of gastric tube (day)	1.29 ± 0.46 (1~2)	2.15 ± 0.82 (1~4)	−4.519	<0.001
Anal exhaust time (day)	2.81 ± 0.41 (2.5~4)	3.90 ± 0.78 (2.5~6)	−6.120	<0.001
Water feeding time (day)	2.19 ± 0.32 (2~2.5)	3.26 ± 0.75 (2~5)	−6.469	<0.001
Solids feeding time (day)	2.77 ± 0.31 (2.5~3.5)	3.76 ± 1.04 (3~7)	−4.453	<0.001
Hospital stay time (day)	5.21 ± 0.29 (5~6)	7.26 ± 4.13(5~20)	−2.425	0.019
Ultrasound- guided catheter drainage rate	0/24 (0%)	2/25 (8%)	Fisher	0.492
Complication			Fisher	*P*
Anastomotic bleeding	0/24 (0%)	1/27 (3%)		1.00
Bile leakage	0/24 (0%)	2/27 (7%)		0.492
Wound infection	0/24 (0%)	3/27 (11%)		0.238
Upper respiratory infection	1/24 (4%)	3/27 (11%)		0.612
Cholangitis	0/24 (0%)	2/27 (7%)		0.492
Stenosis	0/24 (0%)	1/27 (3%)		1.00
Distal lithiasis	0/24 (0%)	0/27 (0%)		1.00
Residual cyst	0/24 (0%)	0/27 (0%)		1.00
Early complications	1/24 (4%)	8/27 (29%)		0.026
Late complications	0/24 (0%)	3/27 (11%)		0.238

## Discussion

The Da Vinci Surgical System was approved by the Food and Drug Administration of America (FDA) in 2000 for clinical use. With the development in the last two decades, now, the robot-assisted technique has become a key component of minimally invasive surgery. However, a child's small abdominal cavity and limited operating space hardly match the large size of the machine, which impedes its application in the field of pediatric surgery to some extent.

Currently, there is no surgical robot designed specifically for children. Many pediatric surgeons have reported modified methods including decreasing the number of robotic arms and increasing the assistant tractor (5–7). In our modification, besides decreasing the number of robot arms, we also reduce the distance between each port. We use only three arms: Nos. 2, 3, and 4 and introduce a quadruple-channel puncture device whose baseplate diameter is about 6 cm. When inserted through the two big channels of the device, the camera port III and operating port IV are placed close to the edge, so that the distance between them can be up to 4 cm. Meanwhile, the other 8 mm incision is made 6 cm on the right side to insert robotic operating port II (Figure 1). In this way, the distance between camera port III and each operating port is about 4 cm; the distance between the two operating ports is about 8 cm. No collision of arms and insufficient clearance are observed in our practice, which may verify that the 6~8 cm distance of each port may not be necessary for children's robot surgery.

Also, we use sequential traction sutures instead of the liver retractor or add an assistant tractor. The Nathanson liver retractor could be too large for small children. Sequential traction sutures include: stitching and lifting the round ligament and the gallbladder fundus with suture before separating and dissecting the cyst; stitching and lifting the common hepatic duct before the hepaticojejunostomy anastomosis. With the tension and traction created by the lifting, creases are minimized and anastomosis is easier to perform as there is less of a chance to miss a stitch. However, the lifting suture is meant to stabilize the surgical view and to offer the operator more control of the surgical field.

The wound appearance was more hidden and beautiful by applying a quadruple-channel puncture device. We make a 2.5–3 cm curved incision along the edge of the umbilical to put the device into the abdomen. It has four channels; two big channels were used to put an 8 mm 3D camera port III and an 8 mm operating port II, and two small channels were used flexibly as an assistant port. In this way, the incisions can be concentrated around the umbilical instead of scattered in the abdominal wall as reported by others (8–11). Furthermore, when we perform jejunojejunostomy outside the abdominal cavity, the baseplate of the device can serve as a container separating the intestinal content and the wound, which may explain why the incidence of wound infection is lower in the R group. A quadruple-channel puncture device can be used for a conventional lap. We also have the experience of laparoscopic surgery by a quadruple-channel puncture device, leading to excellent outcomes of wound appearance. Meanwhile, robotic-assisted surgery, with advantages of flexibility and precision, is effective in dissection and anastomosis in complex surgery. Thus, we decide to combine the two techniques together to invent the trans-umbilical single-site+1 robotic-assisted approach, which has advantages over laparoscopic surgery by a quadruple-channel puncture device alone or robotic surgery alone. Moreover, we can omit the procedure of enlarging and resuturing the umbilical incision, which is more convenient and saves some operative time.

In 1995, Farello first reported the laparoscopic excision of the choledochal cyst (12). In recent 10 years, pediatric surgeons have gradually applied the experience of laparoscopic-assisted procedure to the robot-assisted procedure (5, 13, 14). Due to the Da Vinci Surgical System's advantages of 3D visualization, intuitive instrument control, tremor reduction, enhanced dexterity, and the wristed instruments, the precision of dissection and anastomosis are immensely improved. Though compared to traditional laparoscopic surgery, the median total operative time of robot-assisted surgery was a little longer (217.63 ± 5.90 vs. 199.37 ± 5.13 min, *P* < 0.001), which may be due to device installation, docking, and cosmetically wound suture. However, the R group had shorter mean choledochal cyst excision time and mean hepaticojejunostomy anastomosis time, and volume of blood loss. After the surgery, the patients of the R group had a shorter indwelling time of the gastric tube, anal exhaust time, water feeding time, solids feeding time, and hospital stay time than the L group. The early complications of the R group, such as anastomotic bleeding, bile leakage, and wound infection, are also lower than the L group. In 2020, Chi SQ (5) compared outcomes in robotic vs. laparoscopic-assisted choledochal cyst excision and hepaticojejunostomy in children. The research contained 70 children in the robot group and 70 children in the laparoscopic group, which was the largest series report at present. The conclusion also confirmed that robotic surgery had better ergonomics, less secondary damage, and a lower incidence of early postoperative complications, compared to laparoscopic surgery. It was an effective way to achieve the goal of rapid postoperative recovery.

In conclusion, as the concept of enhanced recovery is continuously developing, robot-assisted surgery will become a new trend in minimally invasive surgery. A resection of the choledochal cyst and a Roux-en-Y hepaticojejunostomy can be performed much more precisely by single-site+1 robotic-assisted surgery. Patients can achieve rapid recovery, and the umbilical incision is more concealed and beautiful. By accumulating the experience of single-site laparoscopic surgery and combining it with a robot-assisted system, the operators can implement the technique in children safely and feasibly.

## Data Availability Statement

The original contributions presented in the study are included in the article/Supplementary Material, further inquiries can be directed to the corresponding author.

## Ethics Statement

The studies involving human participants were reviewed and approved by K2020-06-016. Written informed consent to participate in this study was provided by the participants' legal guardian/next of kin.

## Author Contributions

SL design the study, write manuscript, and assist in surgery. JC design the study, revised the manuscript, assist in surgery, and perform the statistical analysis. KT assist in surgery and collect clinical data. YH and XX collect clinical data. DX perform all robotic surgries, guide, supervise, and finance the study.

## Funding

This work is supported by the Science and Technology of Fujian Provincial (Grant Number: 2021J01386).

## Conflict of Interest

The authors declare that the research was conducted in the absence of any commercial or financial relationships that could be construed as a potential conflict of interest.

## Publisher's Note

All claims expressed in this article are solely those of the authors and do not necessarily represent those of their affiliated organizations, or those of the publisher, the editors and the reviewers. Any product that may be evaluated in this article, or claim that may be made by its manufacturer, is not guaranteed or endorsed by the publisher.
